# High acceptance of artemisinin-based combination therapy for the home management of malaria in rural communities in southwest Nigeria

**DOI:** 10.5281/zenodo.10878717

**Published:** 2014-04-22

**Authors:** Catherine Olufunke Falade, Ikeoluwapo Oyeneye Ajayi, Oyindamola Bidemi Yusuf, Franco Pagnoni

**Affiliations:** 1 Department of Pharmacology and Therapeutics, College of Medicine, University of Ibadan, Ibadan, Nigeria; 2 Department of Epidemiology and Medical Statistics, College of Medicine, University of Ibadan, Ibadan, Nigeria; 3 Evidence for Antimalarial Policy and Access Unit, UNICEF/UNDP/World Bank/WHO Special Programme for Research and Training in Tropical Diseases (TDR), Geneva, Switzerland

## Abstract

**Background:**

Artemisinin based combination therapy (ACT) is the global gold standard for treatment of malaria. In sub-Saharan Africa the majority of malaria cases is treated at home. In rural southwest Nigeria we set out to evaluate the feasibility and acceptability of using artemether-lumefantrine (AL) at the community level to treat acute uncomplicated malaria.

**Materials and Methods:**

Following advocacy and community mobilisation in a rural area in south-west Nigeria, 60 community medicine distributors (CMDs: patent medicine sellers, selected mothers from the community and health-care workers) were trained to recognise the signs and symptoms of childhood malaria and to treat febrile children aged 6–59 months with AL, after ruling out certain danger signs. At the end of one year, the programme was evaluated by conducting a 2-week fever recall survey among caregivers, inspection of CMD records to evaluate caregivers’ adherence to the treatment schedule, CMDs’ performance and the coverage of febrile children with AL. Data was analysed using descriptive statistics.

**Results:**

Based on CMDs’ records, 97.6% (1019/1044) of the children treated with AL received the correct dose. Over half (52.3%) of the children (288/551) whose caregivers participated in the 2-week fever recall survey reportedly received AL from a CMD. Reasons for not receiving AL included non-availability of a CMD [35.7%; 94/263] or drug stock out [28.1%; 74/263]. Of the children treated with AL, 80.2% (231/288) received prompt treatment at the correct dose and for the correct length of time. Ninety-eight percent of the caregivers perceived AL to be effective and none reported severe adverse events.

**Conclusions:**

The use of AL at the community level is feasible and acceptable in the home management of malaria in rural southwest Nigeria. Challenges that must be addressed include avoiding stock outs, ensuring adequate number of CMDs and providing incentives to ensure their availability.

## 1 Introduction

Studies have shown that prompt and appropriate treatment of suspected cases of childhood malaria close to the home can remarkably reduce malaria morbidity and mortality [1,2]. In sub-Saharan Africa, where the disease remains a major public health burden, children’s caregivers and community drug providers are actively involved in treating childhood malaria at home and take affected children to healthcare facilities only if they fail to improve or have severe disease from the onset [3-5]. The different categories of community drug providers include patent medicine sellers, market stall drug sellers, drug hawkers, village health workers and retail pharmacists.

Nigeria changed the drug of choice for uncomplicated malaria from chloroquine to artemisinin-based combination therapy (ACT), with artemether-lumefantrine (AL) as the first option, in January 2005. Through the home management of malaria strategy, the country’s Federal Minis-try of Health is introducing AL for distribution by the informal sector at the community level outside health facilities [6]. However, this has raised concerns such as the relatively high cost of AL and its complicated dosage regimen both of which can lead to poor treatment adherence. Furthermore, the practice of presumptive treatment of malaria, which is practiced in malaria-endemic areas, usually leads to over-prescription of antimalarial drugs with the attendant risk of the selection of drug-resistant parasites [7,8]. Ideally, ACTs should be reserved for cases that have been confirmed in the laboratory as recommended by the World Health Organization. Fortunately, some malaria rapid diagnostic tests (RDTs), which are recommended for accurate diagnosis, especially in rural areas, have shown high sensitivity and specificity and can be reliably performed by community health workers after minimal training [8,9].

This article reports the results of a study in which the feasibility and acceptability of the use of AL at the community level were assessed in rural southwest Nigeria to guide the drug’s deployment in the home management of malaria strategy. The study was part of a multi-country study carried out in Ghana, Nigeria and Uganda, with sup-port from the UNICEF/UNDP/World Bank/WHO Special Programme for Research and Training in Tropical Diseases, in which 59% of 2190 children with fever received ACT from community medicine distributors (CMDs). Coverage varied from 52% in Nigeria to 75% in Ho District, Ghana. A large proportion of children in that study were reported to have received prompt treatment with the correct dose and for the correct length of time [10]. Almost all caregivers perceived ACT to be effective.

## 2 Materials and Methods

### 2.1 Study site and population

The study was conducted in two rural health districts selected by simple random sampling from the eight health districts in Ona-ara local government area (LGA) in southwest Nigeria, previously described by Ajayi *et al.* [5]. Ona-ara LGA was purposively selected for the study based on its successful participation in previous studies conducted in the area. Forty communities were selected from the two districts with selection probability proportional to size. Caregivers who had children 6–59 months of age with fever and CMDs who consented to participate were enrolled in the study. The CMDs comprised patent medicine sellers (non-pharmacist drug shop keepers), facility-based health-care workers and lay mothers (hereafter called “mother trainers”) selected by the communities and trained to distribute AL, deliver treatment guidelines to all house-holds in the community, and provide health education on malaria to community members.

### 2.2 Study design

This was a quasi-experimental study conducted in three phases. Phase 1 comprised formative activities including community entry, field preparation and collection of baseline data. Phase 2 was devoted to training the selected community drug providers, including lay community members, who were to be mother trainers, hereafter referred to as CMDs. They were taught how to recognise the signs and symptoms of malaria, rule out danger signs such as recurrent convulsions, loss of consciousness or protracted vomiting. They also received training on how to distribute AL to febrile children 6–59 months of age and treatment guidelines to all households. The CMDs’ performance, caregivers’ adherence to the treatment schedule and the coverage of febrile children with ACT, one year after AL was first distributed, were evaluated using a 2-week fever recall household survey and analysis of the CMDs’ client registers in Phase 3 of the study. The main outcome measures were the feasibility and acceptability of using AL in the community. Feasibility was evaluated on the basis of caregivers’ and CMDs’ adherence to the treatment schedule, storage of AL by CMDs in their respective homes and coverage with AL. Acceptability of use of AL was evaluated on the basis of CMDs’ use by caregivers of febrile children, the perceived effectiveness of AL by care-givers and caregivers’ satisfaction with treatment outcome.

### 2.3 Sample size

A minimum population of about 40 000 – that of a typical district – was considered adequate for the study [10]. The sample size for the household survey was based on a compliance rate of 52% from a past intervention study on the home management of malaria [5] to provide a precision of ± 5% for the estimated coverage of AL treatment through CMDs, assuming a design effect of two. The minimum number of households to be interviewed was 760. In each household, one consenting caregiver with a child 6–59 months of age that had been febrile in the 2 weeks before the survey was interviewed.

### 2.4 CMD selection, training and supervision

CMDs were selected only if they had lived in the community for at least one year, were trusted and respected by the community, could read, write and keep simple records, were willing to serve, and had obtained consent from their husbands (if they were mother trainers). On average, two CMDs were selected per community in accordance with the size of the population. A 3-day workshop was held for the 60 CMDs selected. It consisted of two days of didactic lectures and demonstrations followed by one day of practical field training. Re-training was carried out after one month and when needed thereafter. CMDs were trained to recognise the signs and symptoms of malaria, rule out danger signs, distribute AL, provide health education to the community and distribute an AL treatment guideline to every household in their community. They were also trained to refer children presenting with non-malarial illness or severe disease to health facilities. Project supervisors visited the CMDs monthly and performed seven unscheduled visits during the one-year intervention period to check drug stocks, drug storage conditions and the CMDs’ registers.

### 2.5 Study drug and drug distribution

Pre-packaged fixed-dose combination of artemether (20 mg) and lumefantrine (120 mg) of different colours was used in this study. Children 6–35 months of age received one tablet twice daily for 3 days (1 × 6; yellow pack), while children 36–59 months of age received two tablets twice daily for 3 days (2 × 6; blue pack) following a presumptive diagnosis of malaria. A detailed explanation of the treatment schedule and the need to administer AL with meals, especially fatty foods was also given. Caregivers administered all doses of AL to their children at home without supervision. AL was purchased through the WHO for the first 6 months. During this period, 1 × 6 and 2 × 6 packs were sold for the equivalent of 20 and 30 US dollar cents, respectively. Prices were fixed by CMDs in consultation with community members to foster community participation and ownership. For the second half [six months] of the intervention year, AL was provided by the Global Fund for Tuberculosis, HIV/AIDS and Malaria through Nigeria’s Federal Ministry of Health. In accordance with the policy of the Federal Ministry of Health AL was then distributed free for the remainder of the study period.

Study drugs were distributed through existing public health centres from where CMDs replenished their stock as necessary. AL was sometimes delivered by the research staff during supervisory visits. Each CMD was provided with a plastic box for the safe keeping of AL and instructed to keep the drug in a well-ventilated area of the home on a raised shelf away from heat, sunlight and out of the reach of children. Health centres were provided with parenteral artemether to initiate treatment for any cases of severe malaria that were referred to them before referral to the nearest secondary healthcare facility.

### 2.6 Other supplies

CMDs were provided with case record forms/registers, adverse event forms, consent forms, referral forms and an AL treatment guideline that had been adapted from a previous study on the home management of malaria using chloroquine [11]. The treatment guideline was a poster in cartoon format and had pictorial illustrations of some common clinical features of uncomplicated malaria and the correct AL dose and regimen for each age group [12]. CMDs were not paid a salary but received a commission equivalent to 30 US dollar cents per pack of AL dispensed as compensation for the time they spent on the assignment. They were also given incentives in the form of transportation fare for drug collection trips, periodic gifts (drinking cups, rice and vegetable oil during festivities), T-shirts with the project logo and certificates of participation.

### 2.7 Data collection tools

The questionnaire used for data collection was designed in English, translated to Yoruba (the local language), back translated to English and tested before use. Information was collected on: 1) treatment-seeking behaviour among caregivers of children who had had fever in the 2 weeks before the survey; 2) CMDs’ availability; 3) the quality of CMDs’ explanations of the AL dosage schedule, possible adverse effects and danger signs; and 4) caregivers’ perception of the effectiveness of AL. Caregivers’ adherence to the correct dose of AL was determined on the basis of their report on the type (1 × 6 or 2 × 6 and colour) of the AL pack dispensed to them, the number and distribution of daily tablets, the duration of the treatment and inspection of the pack of AL, if available, for leftover tablets. The CMDs’ registers provided information on treatment coverage, the characteristics of the children treated, the dose of AL dispensed and the timeliness of caregivers’ visit to the CMDs. The supervisors’ records provided information on the stocking of AL by CMDs and on how AL was stored.

### 2.8 Data analysis

The data collected were entered into EPI Info version 6.02 (Centres for Disease Control and Prevention, Atlanta, GA, USA) and analysed using SPSS version 11.0 (SPSS Inc., Chicago, IL, USA). The data were analysed using descriptive statistics such as median, proportions and percentages.

### 2.9 Ethical clearance

The Oyo State Ministry of Health Ethical Review Committee and the WHO Ethics Review Committee provided ethical approval for the study. Informed consent was also obtained from community heads, household heads and caregivers who participated in the study.

## 3 Results

Out of a total of 60 CMDs, 47 (78.3%) were mother trainers, 8 (13.3%) were healthcare workers and 5 (8.3%) were patent medicine sellers. Six CMDs dropped out of the study (attrition rate: 10%). Attritions occurred because of death (1 CMD), marriage, or relocation to join family members or to the city for trade (5). The demographic characteristics of the CMDs are shown in Table 1.

**Table 1.** Demographic characteristics of the community medicine distributors (CMDs) involved in the study.

Characteristic	Frequency (N=60)	%
*Sex*		
Male	4	6.7
Female	56	93.3
*Age, in years*		
20-29	13	21.7
30-39	23	38.3
40-49	16	26.7
50-62	8	13.3
*Type of CMD*		
Mother trainer	47	78.3
Patent medicine seller	5	8.3
Health worker	8	13.3
*Education*		
Primary	32	53.4
Secondary	14	23.3
Post-secondary	14	23.3
*Marital status*		
Single	3	5.0
Married	53	88.3
Widowed	4	6.7
*Occupation*		
Trader	26	43.3
Farmer	14	23.3
Community health extension worker	5	8.3
Patent medicine seller	5	8.3
Missionary	3	5.0
Trained nurse	3	5.0
Teacher	2	3.3
Traditional birth attendant	2	3.3

### 3.1 CMD performance and adherence

Of 1044 fever episodes in children 6–59 months of age that were treated with AL as recorded in the CMDs’ register during the study period, 53.0% occurred in girls. About half (49.9%) of the children who had fever were aged 6–35 months, and 50.1%, were aged 36–59 months. Correct dose of AL was received by 1019 (97.6%); 11 (2.1%) and 12 (2.3%) of the younger and older children were given an excessively high dose, respectively. Of 288 caregivers who reported during the survey that their children received AL from CMDs, 264 (91.7%), 241 (83.7%) and 240 (83.3%) reported that the dosing schedule, the possible adverse effects of the drug and the danger signs, respectively, had been explained to them by the CMD. It took a median of 5 minutes (range: 1 to 120) for caregivers to walk from their homes to visit a mother trainer and collect AL, and most caregivers (87.5%) were able to find the CMDs during their first visit. All CMDs were found to have stored AL as recommended at supervisory visits. Eighteen (30.0%) of them ran out of AL for 1 to 3 days during the study period. The median number of times CMDs ran out of stock was 2 (range: 1 to 14 times). Reasons provided for this included the following: the CMD was too busy to replenish the stock (38.8%); AL was unavailable at the health-care facility (33.3%); the health facility worker had refused to replenish the stock (16.7%), or the CMD did not realise the stock was low (11.8%). Whenever CMDs ran out of AL stock, they usually went to the health centre to re-stock, collected AL from fellow CMDs, referred the caregiver to a health centre, waited for research staff to replenish the stock, asked the caregiver to buy other antimalarials, or resorted to dispensing cotrimoxazole and paracetamol.

### 3.2 Treatment of febrile children

The parents or caregivers of 551 children participated in the 2-week fever recall survey. Reportedly, 288 (52.2%) children received AL from CMDs: 165 (57.3%) from mother trainers, 101 (35.1%) from patent medicine sellers and 22 (7.6%) from health workers. Further details of treatment coverage are shown in Table 2. Reasons given by the caregivers of 263 children who did not receive AL included non-availability of the CMD [94 (35.7%)], the CMD had run out of AL stock [74 (28.1%)] or was not interested in using the drug [12 (4.6%)]. Eleven (4.2%) caregivers said they did not know the CMD still distributed AL; 9 (3.4%) used other drugs, and 3 (1.1%) said they could not afford AL. No response was given by 60 (22.8%) caregivers. Other drugs that were used for treating febrile children by caregivers included other antimalarials, antibiotics and herbal preparations.

**Table 2.** Characteristics of febrile children treated with artemether-lumefantrine (AL) obtained from community medicine distributors (CMDs), as well as their caregivers.

Characteristics	Frequency	%
*Age, in months*		
6 -11	17	5.9
12 – 23	49	17.0
24 – 35	69	24.0
36 – 59	153	53.1
*Sex*		
Female	154	53.5
Male	134	46.5
*Education (of caregiver)*		
None	80	27.8
Primary	155	53.8
Secondary	53	18.4
*Marital status (of caregiver)*		
Married	256	88.5
Not married	30	10.5

### 3.3 Caregiver adherence

Analysis of the CMD’s registers showed that 767 (73.5%) of 1044 febrile episodes were reported to the CMDs within 24 hours after the caregiver noticed the first symptoms in the children. Of the 288 children who reportedly received AL at the 2-week fever recall survey, 278 (96.5%) were given the first dose within 24 hours of the onset of symptoms. Caregivers gave 256 (89%) children the correct dose for the right length of time and treated 231 (80.2%) children both promptly and correctly. Nineteen (6.6%) caregivers had leftover tablets in the AL packs used because the child was no longer ill (13) or did not improve (5), while one caregiver said she was saving the remaining drug for future use. About 60% (332/551) of the respondents in the 2-week fever recall survey had copies of the treatment guideline. Of the 288 (87.5%) whose children had received AL, 252 had the guideline; 95.3% of them reported having understood it and 86.9% claimed having referred to it.

### 3.4 Safety and perceived effectiveness of AL

Of the 288 caregivers whose children were reported to have received AL at the 2-week fever recall survey, 280 (98%) reported that the children recovered after using the drug and perceived AL to be effective. The eight caregivers whose children did not recover after receiving AL reported seeking care elsewhere. Ten (3.5%) caregivers reported mild adverse events such as rashes, vomiting worms, ‘yellow urine’, cough and tiredness. Five such events were reported to mother trainers, who referred them to a health facility.

## 4 Discussion

In this study, caregivers and CMDs showed good adherence to AL use at the community level. The 52% treatment coverage recorded in this study – well above the 33% and 47% coverage estimates for Tanzania and the Gambia, respectively – is impressive, especially since AL was being introduced into the community for the first time [13,14]. The high treatment coverage found and the prescription of the correct dose of AL by most caregivers and CMDs are evidence of the acceptability of AL and of the feasibility of using it within the context of the home management of malaria.

Adherence to full treatment with ACT in the home management of malaria strategy has been a major concern, especially because of the twice daily dose of AL for 3 days [15,16] against the background of poor adherence to chloroquine despite its simpler dosing [3-5]. Thus, the fact that 97% of the CMDs and 89% of caregivers adhered to AL provides evidence to support the use of AL at the community level. Levels of adherence by caregivers in this study were similar to those found in Uganda [17,18] but higher than in Zambia (61%) [15] and southern Sudan, (78.8%) [16], where studies were conducted in refugee and internally displaced populations. Effective training, supervision, provision of a treatment guideline, absence of serious adverse events, good community mobilisation and commitment are additional factors that could have contributed to the high treatment adherence and acceptance recorded in this study. In previous intervention studies, training was found to have positively influenced adherence to chloroquine in the home management of malaria [19,20].

Of the caregivers who gave AL to febrile children suspected of having malaria, 98% felt that the drug had been effective. Some of the children who did not receive AL were reportedly treated with antibiotics or other drugs. This is noteworthy because it suggests that some caregivers and CMDs suspected that the children had a non-malarial febrile illness such as pneumonia, dysentery or meningitis, whose symptoms resemble those of malaria. In febrile children these illnesses, especially pneumonia, should always be suspected [21-23]. In a case series study, Källander *et al.* [21] reported that in Uganda, delay in seeking care for pneumonia had contributed to a large proportion of deaths among children aged 1–59 months, 52% of whom had received antimalarial drugs.

In this study, the high perceived effectiveness of AL is consistent with the results of previous studies in which AL was shown to be highly effective, whether given with [24,25] or without supervision [18]. The fact that CMDs were within walking distance of caregivers enhanced access to AL. The 10% dropout rate among CMDs in this study was less than in previous studies [26,27]. Salako *et al.* [26] and Ajayi *et al.* [20], who conducted research in southwest Nigeria, recorded dropout rates of 49% and 24.2%, respectively, among trained drug distributors. CMDs in this study, unlike those in earlier studies, received financial incentives. The importance of training and supervision of CMDs, of providing incentives and of recognising the work of community volunteers has been high-lighted in several studies [20,28,29].

A significant proportion of children in the 2-week fever recall survey did not receive AL because no CMD was available or AL was out of stock. These are important is-sues that must be addressed if AL use in the home management of malaria is to succeed. Offering incentives for CMDs, increasing the number of CMDs and ensuring a regular drug supply may overcome these barriers.

## 5 Conclusions

AL was found to be well accepted, effective and safe when used at the community level in Ona-ara LGA in southwest Nigeria. Investing to improve and sustain treatment adherence, providing incentives to CMDs and strengthening the health system should be integral to the scaling up of ACT use for the home management of malaria in Nigeria and beyond.
